# Immunological Aspects of Acute and Recurrent Herpes Simplex Keratitis

**DOI:** 10.1155/2014/513560

**Published:** 2014-09-07

**Authors:** Jacek Rolinski, Iwona Hus

**Affiliations:** ^1^Chair and Department of Clinical Immunology, Medical University of Lublin, Chodzki 4a, 20-093 Lublin, Poland; ^2^Department of Clinical Transplantology, Medical University of Lublin, Staszica 11, 20-081 Lublin, Poland

## Abstract

Herpes simplex keratitis (HSK) belongs to the major causes of visual morbidity worldwide and available methods of treatment remain unsatisfactory. Primary infection occurs usually early in life and is often asymptomatic. Chronic visual impairment and visual loss are caused by corneal scaring, thinning, and vascularization connected with recurrent HSV infections. The pathogenesis of herpetic keratitis is complex and is still not fully understood. According to the current knowledge, corneal scarring and vascularization are the result of chronic inflammatory reaction against HSV antigens. In this review we discuss the role of innate and adaptive immunities in acute and recurrent HSV ocular infection and present the potential future targets for novel therapeutical options based on immune interventions.

## 1. Introduction

Typically, individual humans respond to a virus infection in different ways. It is particularly characteristic of chronic viral infections that clinical expression is highly variable. We do not fully understand the reasons for the varying outcome of virus infections in different persons but certainly multiple factors are involved. Herpes simplex virus (HSV) is a smart pathogen, which displays both lytic and latent modes of interaction with its natural human host. The incidence of HSV infection increases with age and anti-HSV antibodies are found in about 88% of individuals at the age of 40 years [1]. During its evolution, HSV has developed a multitude of strategies to hide from immune evasion and counterattacks against the host cell during the reactivation phases.

Herpes virus keratitis (HSK) is the second leading cause of blindness, after cataract, in developed countries, mainly due to its recurrent nature. There are now eight recognized human herpes viruses: herpes simplex virus type 1 (HSV-1), HSV-2, varicella-zoster virus (VZV), cytomegalovirus (CMV), Epstein-Barr virus (EBV), human herpes virus 6 (HHV-6), human herpes virus 7 (HHV-7), and human herpes virus 8 (HHV-8) and the most often cause of keratitis is HSV-1, responsible for 78%–98% of cases [2]. Humans are the only natural host for herpes simplex virus types 1 and 2. Based on the type of inflamed tissue, the herpetic ocular disease could be classified as blepharitis, conjunctivitis, intraocular inflammation, retinitis, and epithelial keratitis that are typical sites for primary infection and stromal keratitis being a most common form of recurrent disease. Primary infection occurs usually early in life and can be asymptomatic or symptomatic. Corneal epithelium is one of the major sites of primary infection [3]. HSV enters oral mucosa, the eye, and the skin through small lesional cuts and abrasions to reach epithelial cells which represent the primary targets of HSV. The infection gate may be ocular surface by droplet spread or the virus might be transferred from the other sites, most often from the mouth. After the initial exposure, the virus replicates in epithelial cells, causing different grades of inflammatory manifestations, from only mild to ulcerative lesions. After primary infection of skin or mucosa, HSV goes into the sensory nerve endings and is conveyed by retrograde axonal transport to the dorsal root ganglion, where the virus develops lifelong latency. During latency, the virus does not generally replicate and does not damage neurons. Intermittent shedding of trigeminal ganglion-based virus can be detected in tears and saliva of adult humans without apparent clinical disease [4]. Immune control of viral infection and replication occurs at the level of skin or mucosa during primary or recurrent infection and also within the dorsal root ganglion, where immune mechanisms control latency and reactivation [5]. The study of anti-HSV immune responses as well as the corresponding viral countermeasures is important to our understanding of antiviral immunity and pathogenesis of herpes simplex keratitis. The immune response against HSV involves both innate and adaptive immune mechanisms. The innate antiviral response, mostly the production of type I interferons (IFN-*α* and IFN-*β*), is thought to play a pivotal role in determining the outcome of an HSV infection. While IFN-*γ* is of crucial importance in maintaining the virus in a latent state and preventing reactivation, in vitro studies showed that type I IFNs may be of importance during establishment of latency [6]. In addition, natural killer (NK) cells, plasmacytoid dendritic cells (pDCs), and macrophages have been shown to contribute to the innate immune responses to HSV. NK cells play an important role both in cytokine production and in recognition and killing of virally infected cells. NK cells producing IFN-*γ* and macrophages/microglia producing TNF-*α* exert a role in maintaining HSV-1 latency in trigeminal ganglia [7]. Also, plasmocytoid dendritic cells (pDCs), whose primary role involves type I IFN production in vivo, are highly involved in antiviral defense. pDCs are a functionally distinct subset of DCs and were originally identified as natural IFN producing cells, as they are the major producers of IFN-*α* in vivo. Studies by Mott and Ghiasi revealed the important role of CD11c+CD8*α*+ DCs that enhance the latency of HSV-1 [8]. Their recent studies revealed a negative function of CD8*α*+ DCs that contribute to T cell exhaustion in the presence of viral RNA molecule, latency-associated transcript (LAT), leading to larger numbers of latent viral genomes in the trigeminal ganglion of intraocularly infected mice and enhanced recurrences [9, 10]. LAT gene is required for wild-type reactivation of herpes simplex virus. Upon infection of neurons, HSV-1 is capable of both inducing and inhibiting apoptosis. In mice, infection of the trigeminal ganglia results in virus replication and neuronal cell death in some nerves. However, trigeminal ganglion neurons remain resistant to apoptosis during HSV latency even in the continual presence of cytotoxic CD8+ immune cells. LAT is viral factor that has been implicated in this protection from apoptosis. Trigeminal ganglion neurons, infected with a LAT-expressing HSV-1, are protected from apoptosis once they become latent. In rabbit trigeminal ganglia, extensive apoptosis occurred with LAT(−) virus but not with LAT(+) viruses [11].

In addition, the adaptive immune response has been shown to play important roles in disease progression, latency, and control of virus spread. While earlier studies have elucidated a role for antibody-mediated protection against infection, a growing body of literature highlights the crucial role of cellular immunity against HSV. HSV-1 specific CD8+ T cells, playing a pivotal role in this process, employ both lytic granule-dependent and IFN-*γ*-dependent effector mechanisms in maintaining HSV-1 latency and inhibiting its reactivation [12–14]. Also HSV-1 specific CD4+ T cells are engaged in HSV-1 clearance from dorsal root ganglions possibly via nonlytic mechanism [15] and local control of infection [16]. Intermittent reactivation leads to anterograde transport of virus particles and proteins to the skin or mucosa, where the virus is shed and/or causes disease. Most ocular diseases are thought to represent recurrent HSV disease following the establishment of viral latency in the host, rather than a primary ocular infection. Chronic visual impairment and visual loss are caused by corneal scarring, thinning, and vascularization connected with recurrent HSV infections. The extension of the lesions may vary from only mild to necrotizing stromal keratitis. Bilateral disease, recurrent infections, and corneal scarring occur more often in immunocompromized patients [3]. In immune-suppressed individuals, like organ transplant recipients, patients receiving chemotherapy, or patients with HIV, recurrent infections might be life-threatening. Disease severity and extension of lesions as well as latency and recurrence depend on viral genes encoded by specific strains and host immune system both innate and adaptive.

## 2. Dendritic Cells as the First Cells Interacting with HSV

Dendritic cells (DC) as antigen-presenting cells (APC) located at the border zones of the body and the environment have been shown to play a crucial role as one of the first cells interacting with HSV beside epithelial cells, on one hand, and as important controllers of the viral spreading on the other hand [1]. The studies on the role of DC in primary HSV infection are limited and they brought divergent results that may result from the different functions attributed to different DC populations. Frank et al. showed that CD11+ DCs are required after HSV-1 corneal infection to orchestrate an innate immune response by directly and indirectly inducing production of chemokines attracting NK cells and inflammatory monocytes engaged in virus clearance from the cornea [17]. Also, CD11c− plasmocytoid dendritic cells known for their high antiviral activity were found to rapidly produce large amounts of IFN-*α* and IFN-*β* after exposure to HSV [18]. Studies by Bryant-Hudson and Carr demonstrated that CD11c+ dendritic cells expressing programmed death 1 ligand (PD-L1) are important for antiviral defense during acute HSV-1 infection, since blockade of PD-1: PD-L1 signaling decreases the activation of dendritic cells resulting in an increased viral load infection [19]. The exact role of different subpopulations of DC in anti-HSV innate and adaptive responses remains to be clarified. The passage of HSV antigens to lymph nodes usually occurs in DC and HSV can inhibit DC maturation. As the other effective defense strategy, HSV-1 induces apoptosis of attacking DC and the downregulation of the expression of costimulatory molecules, such as CD80, CD86, CD40, the adhesion molecule CD54 (ICAM-1), chemokine receptors CCR7 and CXCR4 on mature DC, and major histocompatibility class (MHC) I molecules [1].

## 3. Toll-Like Receptor: Mediated HSV Recognition

The recognition of pathogen molecular patterns by toll-like receptors (TLR) is thought to be crucial for the initiation of the primary innate and later adaptive, immune response. The expression of TLR was found in epithelial corneal cells; however the role of TLR in the initiation and control of HSV infection is still not clear. Upregulated mRNA levels for TLR 4, 7, 8, and 9 in human cornea with active keratitis and upregulated TLR7 expression in cornea with nonactive keratitis as compared to the normal cornea suggest the role of these receptors cells in the HSV-1 infection [20]. There are suggestions that TLR2 plays a role in the induction of an immunopathological response in the cornea since, in mice lacking TLR2, keratitis lesions were significantly diminished [21]. In TLR4 knockout mice, more rapid and severe lesions were observed, suggesting that TLR4 ligation might serve to protect from severe inflammatory response. TLR (with the exception of TLR3) uses the adaptor molecule MyD88 to initiate intracellular signal transduction. Mice lacking the adapter molecule MyD88 were resistant to lesion development, but such animals were also unable to control infection, succumbing to lethal encephalitis [21]. Much attention has been paid to TLR9 that recognizes CpG motifs of viral genomes including HSV-1. TLR9 is abundantly expressed in cultured human endothelial corneal cell and its ligation initiates signaling that elicits antiviral immune responses to HSV-1 infection, including production of inflammatory cytokines, especially type I interferon (IFN) and chemokines, as well as inducting the host adaptive immune response [22, 23]. However HSV-1 uses TLR-mediated NF*κ*B activation for its own replication [24] and purified HSV DNA, such as synthetic CpG DNA, the ligand for TLR9, was shown to induce ocular neovascularization [25]. From one side HSV-1 DNA recognition by TLR induces the mechanisms of immune defense aiming at virus clearance from the cornea; however by initiating innate immune response, TLR ligation might also exacerbate inflammatory process leading to cornea destruction and there are hypotheses that treatment with TLR antagonists might be of some clinical benefit, especially that glucocorticosteroids used to reduce inflammation in HSK are suggested to diminish TLR3 signaling [26]. This approach seems to be attractive, especially in the context of studies of Conrady et al. showing the elicitation of effective anti-HSV-1 immune response induced by DNA sensor IFI-16 (p204/IFN inducible protein 16) ligation despite the loss of TLR signaling [27]. So, probably, manipulation of the TLR ligand response could provide a means to modulate stromal keratitis lesions; however future studies are needed to define the role of TLR in herpetic keratitis initiation and control.

## 4. HSV-Nonspecific and Specific Immune Responses during Acute Infection

The primary HSV exposure induces rapid infiltration of innate response cells, mainly neutrophils, macrophages, and NK cells. The role of NK cells and macrophages is thought to be crucial in clearance of virus after the initial exposure [28]. NK cells could directly lyse HSV-infected cells and indirectly inhibit HSV proliferation by IFN-*γ* secretion [29, 30]. Macrophages control viral replication during primary infection by secreting nitric oxide (NO), TNF-*α*, and IFN-*γ* [31, 32] and play a major role in recruitment of the innate response cells as well as in the initiation of adaptive T cell mediated immune response [33]. On the other side, they may contribute to aggravate the inflammation resulting in corneal damage [33]. Innate immune cells secrete various proinflammatory cytokines such as interferons (IFN-*α*, IFN-*β*, and IFN-*γ*), IL-1*α*, IL-1*β*, TGF-*β*, TNF-*α*, IL-6, IL-8, IL-12, and IL-17 [28, 30]. Type I interferons are crucial in limitation of HSV replication in the cornea as well as the systemic spread of infection [34]. The sources of IFN-*α* are HSV-infected and adjacent uninfected corneal epithelial cells as well as TLR-activated macrophages [34]. Type I IFNs production is induced by TLR ligation but also, in TLR-independent manner, by activation of IFI-16 that was recently demonstrated in mice by Conrady et al. [27]. IFI-16 has been shown also to induce IFN-*α* driven production of CCL2, chemokine responsible for recrutation of inflammatory monocytes to the infection site. Mice deficient in the A1 chain of the type I IFN receptor (CD118−/−) are extremely sensitive to HSV ocular infection that correlates with a loss of CD4+ and CD8+ T cell recruitment and aberrant corneal production of chemokine trafficking adaptive response cells, suggesting the role of type I IFNs not only in innate but also in adaptive response [34]. In many studies also an important role of IFN-*γ* in HSV clearance in early phase of infection has been shown [35–37]. IFN-*γ* neutralization during preclinical phase of HSV infection, when replication is still present in the cornea, results in increased tissue damage presumably caused by the virus indicating early protective role of this cytokine [38]. The initial source of IFN-*γ* after HSV infection is innate cells, than starting from day 7, the main secretion is by Th1 cells [35, 39]. Important producers of IFN-*γ* during the acute phase of viral infection are *γ*/*δ* T cells that represent a small population of immune cells, but play an indispensable role in host defenses against HSV-1 infection. Increased numbers of *γ*/*δ* T cells have been observed in animal models of HSV-1 infection. In murine herpetic keratitis *γ*/*δ* T cells were observed in the corneal stroma from 1 to 8 days after infection. Together with neutrophils in the early phase of infection, *γ*/*δ* T cells may play an additional role in protecting the cornea against incoming pathogens [40]. In another study, *γ*/*δ* T cells limited severe HSV-1-induced epithelial lesions and greatly reduced mortality by preventing the development of lethal viral encephalitis. The observed protection resulted from *γ*/*δ* T cells cell-mediated arrest of both viral replication and neurovirulence [41].

Recent studies investigated the role of one of the newly discovered cytokines, IL-17 in the HSV acute ocular infection. IL-17 was found to play a critical role in autoimmune phenomena and it has also strong proinflammatory properties, mainly due to enhancing neutrophils influx to the inflammation site, not directly but by induction of specific cytokines and chemokines recruiting neutrophils [42–44]. It also acts as neutrophil survival factor and induces the production of tissue damaging factors like matrix metaloproteinasas and oxyradicals [45]. Also in HSV infection, IL-17 acts by indirect promotion of the recruitment of neutrophils by the induction of chemokines production, mainly MIP-2, by fibroblasts, since active IL-17 receptors (IL-17R) are present on cultured stromal corneal fibroblasts [46]. Absence of IL-17 results in the decrease in IL-17 proinflammatory mediators and reduced neutrophil migration. Though neutrophils are the major component of the inflammatory infiltrate in HSV-1 infected corneas, recent studies by Frank et al. confirmed earlier suggestions [47, 48] that neutrophils, in contrast to NK cells and macrophages, are not essential for clearing HSV-1 from the infected cornea [17]. Since neutrophil migration in HSV infection is associated with enhanced corneal opacity, these observations suggest the role of Th17 in tissue damage. IL-17 is produced in the early phase of infection, rapidly after HSV-1 exposure [43] and its source in the early phase of infection is innate cells, mainly *γ*/*δ* T cells [38]. Though both cytokines have proinflammatory properties, IFN-*γ* negatively affects IL-17 production.

During primary HSV infection, antigen presenting cells, like Langerhans cells, present HSV-1 antigens to T cells. T cell-mediated delayed type hypersensivity (DTH) is supposed to be important for elimination of the virus [28]; however elevated DTH responses are also associated with greater corneal pathology [49–51]. CD8+ T cell-driven immune response is required both to eliminate virus more efficiently from the cornea [52] and to prevent virus transmission outside of the cornea [14, 53]. From the other side, cytotoxic T cells may be connected with more severe course of keratitis [28], with the role of CD4+ cells in acute infection being less clear. Immune response to primary HSV exposure is very complex and protection of infection spread is not dependent on one cell type or cytokine [33].

## 5. Immune-Mediated Complications of Recurrent HSV Infection

Acute HSV epithelial keratitis usually resolves after 1-2 weeks. After the clearance of primary infection, the virus stays in latent form for the life of the host in the nervous tissue, especially sensory neurons of dorsal and trigeminal ganglia. Most recently, cornea has been also proposed as a site of HSV-1 virus latency [54]. Different factors, like hormonal changes, fever, psychological stress, and ultraviolet exposure, may lead to infection reactivations that are predominantly caused by the same strain; however infection with a new strain is also possible [28]. Stromal keratitis occurs in about 25% of people after epithelial keratitis [55]. Mechanisms of indolence and recurrence are still not fully explained. Recurrent herpetic disease is mediated mainly by T cells that both protect against herpetic disease and may potentiate inflammatory reaction. Since ocular morbidity results from recurrent infections, of key significance is to recognize the factors contributing to the recurrent nature of the infection. In this context, a relationship between both virus strain and host genes and severity and recurrence of the infection has been studied. Neither specific viral strains connected with the severity of infection or its recurrence nor the relation between the site of infection and the specific strain was identified. Similarly, host genes concerning both innate and adaptive immune response are thought to influence the course of infection; however still a little is known about host genetics in HSV [56]. The pathogenesis of herpetic keratitis is not fully understood; according to current knowledge corneal scarring and vascularization are the result of the chronic inflammatory reaction against HSV antigens and theory on autoimmunity induced by the infection was not confirmed [29]. Some of the cytokines and chemokines most highly produced by cytotoxic T lymphocytes (CTL), like IFN-*γ*, TNF-*α*, lymphotoxin-*α*, and RANTES, can have multiple antiviral effects on infected cells and the cells around them, including purging of virus from infected cells without killing the cell. This is particularly important for viruses like HSV, which infects nonrejuvenating cells such as nerve cells. It has been found that prostaglandin A might be responsible for recurrent infections by depressing of antibody dependent cellular cytotoxicity (ADCC) and production of IL-2 and low IFN-*γ* [57]. Patients with frequent recurrences have lower levels of INF-*γ* and IL-2 [58, 59]. Herpetic keratitis is Th1 cell dependent and Th1 cytokines play a key role in both inflammation initiation and progression with IFN-*γ* regulating the process [60]. Among the other proinflammatory cytokines, IL-12p35 that promotes IFN-*γ* production is important for late HSK progression [61]. Also IL-17, except its role in acute phase of HSV ocular infection, plays an important role in aggravating the late phase of inflammation. It is produced mainly by sensitized *α*/*β* Th17 cells. This late production of this cytokine is explained by delayed upregulation of IL-6 and TGF-*β*, cytokines promoting the differentiation of naïve T cells into IL-17 producing Th17 cells, and the corneal expression of CCL20 chemokine, recruiting Th17 to inflammation sites [38]. IL-17 was found in mice and human to stimulate fibroblasts to produce chemokines that affect HSK development [62], IL-6, and IL-8 [30] as well as proangiogenic factor VEGF-A [63]. It also increases chemoattractant, CXCL1/KC production [63]. Both CXCL1/KC and IL-8 are essential for the recruitment of PMN. Not only does neutrophil influx contribute to destructive lesions by serving as an activator for HSV-specific T cell-mediated inflammatory responses [35, 64], but also neutrophils have been identified as a source of VEGF-A [65, 66] as well as metaloproteinases, enzymes degrading VEGF-A soluble receptor that blocks its activity [63] contributing to corneal stroma neovascularization. In vitro experiments by Suryawanshi et al. showed that IL-17A not only was stimulatory for VEGF-A gene expression, but also had the opposite effect on sVEGFR-1 expression, suggesting that the effect of IL-17A on angiogenesis not only is inducing VEGF-A but is rather a combined effect on the VEGF-A/sVEGFR-1 axis. The authors further showed that the severity of HSK lesions is diminished in mice lacking IL-17R [63]. In a mouse model of recurrent HSK, Xia and colleagues found that Th17 cells were upregulated in both cornea and draining lymph nodes. Systemic administration of anti-IL17 antibody resulted in diminished corneal opacity, neovascularization, and reduction of CD4+ cell infiltration [67]. These results suggest that strategies targeting IL-17 might be a new valuable therapeutical option for chronic keratitis [38]. In contrast, Th2 cytokines can ameliorate HSK [51]. CD25+ T regulatory cells (Treg) can suppress the inflammatory process, so Treg depletion is associated with elevated HSV-specific CD4+ T cell responses [68] and more pronounced lesions [29]. In the mice model, Veiga-Parga and colleagues showed that the lesions became more severe when Treg depletion was begun in the clinical phase of the disease [69]. In HSK, where tissue damage results mainly from chronic inflammatory process and the replicating virus is minimal or absent, the beneficial effect of Treg seems to be especially relevant [69].

The role of CD8+ T cells in immune surveillance of cornea is much less explained. After the clearance of virus, a small percentage (between 5 and 10%) of effector CD8+ T cells differentiate in heterogeneous subpopulations of memory CD8+ T cells with different function, phenotype, and tissue location. Three main populations were distinguished: tissue resident memory CD8+ T cells (TRM), effector memory CD8+ T cells (TEM), and the central memory CD8+ T cells (TCM). Previous studies [70] suggest that TCM are capable of mounting stronger proliferative responses following reinfection. However, the tissue-specific homing of TEM cells permits them to reside in sites of potential viral infection, such as the skin and mucosae. These resident memory T cells (TRM) are sequestered from the circulation and provide rapid protection against viruses such as HSV in skin, where they localize with a unique dendritic morphology and undergo slow surveillance of the tissue. This is in contrast to CD4+ TEM, which continue to migrate through the nonlymphoid tissues rather than being sequestered in the peripheral tissues, and also differs from the CD8+ and CD4+ TCM, which migrate largely through the lymphoid organs (spleen and lymph nodes). Resident memory CD8 T cells (RM) remain localized in the epidermis in skin after HSV infection. Recently Khan et al. presented the new concept of segregation of HSV-1 specific memory T cells into CD8+ memory T cells specific to asymptomatic and symptomatic viral epitopes (so-called asymptomatic and symptomatic memory T cells) developing within the widely known TEM, TRM, and TCM populations [71]. According to the authors, symptomatic and asymptomatic epitope stimulations are among the major factors influencing the development of different antigen specific memory CD8+ T cell populations after the infection, with asymptomatic memory T cells that protect against herpes infection and symptomatic ones that are responsible for immunopathology and may even lead to herpetic disease exacerbation. By this concept, corneal lesions caused by recurrent HSV infections are not caused directly by virus or by autoreactive or bystander T cells, but by a dominance of immunopathological T cell responses specific to symptomatic HSV-1 epitopes over immunoprotective T cell responses specific to asymptomatic HSV-1 epitopes [71]. These results might be of special value for the future studies on anti-HSV-1 vaccines development indicating the exclusion of symptomatic epitopes from vaccines because they may exacerbate, rather than cure, recurrent infectious.

## 6. Experimental Immune-Based Therapeutic Strategies

Actually, there are two main therapeutic strategies used to prevent visual impairment and blindness associated with chronic HSV keratitis [55]. One is aimed against virus itself and the second is to suppress host processes responsible for corneal damage, like chronic inflammation and angiogenesis. Traditionally in the prevention of HSV keratitis recurrences, antiviral agents are used and infections are treated with topical antiviral drugs and corticosteroids. Long-term low dose oral acyclovir reduces recurrent ocular disease by approximately 45% [28]. Because of incomplete protection, there is an urgent need for the development of new therapeutical methods to reduce morbidity. An example of a new promising antiviral drug is manzamine A, marine-derived *β*-carboline alkaloid, that inhibits viral replication and infection in human corneal cell line [72]. Recently, along with new insights in the disease pathogenesis, novel methods based on immune intervention are under development. Since HSV keratitis is a Th1 dependent inflammation and CD4+ Th1 cells are the main contributors to the corneal destruction, one new promising approach is to modulate Th responses. It was found that HSK can be abrogated by CD4+ effector cell depletion, Th1 cytokines neutralization, or T regulatory cell expansion.

Since the immunopathological lesions are thought to be primarily orchestrated by CD4+ T cells, mainly Th1 cell, and to a lesser extent Th17, reducing the number or diminishing the activity of effector cells is a potentially valuable approach to reduce HSK severity. Rajasagi et al. studied the value of galectin 1, an endogenous lectin on ocular disease caused by HSV-1. Galectin 1 (Gal1) is an immune response modulator that controls the proliferation of effector T cells, blocks the production of proinflammatory cytokines, and increases production of anti-inflammatory cytokines like IL-10. Gal1 administration significantly diminished SK lesion severity and neovacularisation by decreasing the influx of CD4+ cells and innate cells like neutrophils and decreasing the production of proinflammatory cytokines [73]. Resolvin E1 (RvE1), an endogenous lipid mediator that was shown to promote resolution in several inflammatory disease models, was also found to be efficient in controlling ocular disease caused by HSV. RvE1 topical administration markedly diminished corneal lesion severity and neovascularization in experimental mice model. RvE1 therapy decreased the influx of effector CD4+ T cells and neutrophils as well as the production of proinflammatory cytokines and molecules involved in ocular neovascularization [74]. Another endogenous-lipid mediator with anti-inflammatory proresolution properties, neuroprotectin D1 (NPD1), used as topical therapy in the studies of Rajasagi et al. demonstrated the high efficiency against ocular disease caused by HSV, markedly reducing SK lesion severity and corneal neovascularization. It was associated with decreased influx of effector CD4+ T cells and neutrophils and production of proinflammatory cytokines, chemokines, and proangiogenic molecules [75].

Another approach was to use synthetic molecules such as 2,3,7,8-tetrachlorodibenzo-p-dioxin (TCDD), an agonist of the aryl hydrocarbon receptor (AhR). TCDD decreased the proliferation and causes apoptosis of Foxp3−CD4+ T cells having no effect on Foxp3+CD4+ Tregs. The consequence was an increase in the ratio of Tregs to T effectors resulting in a reduction of inflammatory responses. A single TCDD administration given to the mice after the disease process had been initiated and significantly reduced the severity of herpes keratitis lesions [76]. Since Foxp3+CD4+ Treg responses might be beneficial by minimizing the tissue damage caused by chronic inflammatory response [68], expanding Treg and boosting their function might constitute another promising option to control HSK.

Reddy at al. [77] demonstrated interesting data on successful management of corneal damage caused by HSK using combination therapy with Gal-9, which inhibits effector T cell function and monoclonal antibody to tumor necrosis factor receptor superfamily member 25 (TNFRSF25), which expands and activates Tregs. This combination therapy provided more effective lesion control than achieved by treatment with one of them [77].

The next strategy is based on targeting costimulatory or coinhibitory signals of CD4+ T cell activation. In experimental studies on mice model, blockade of important costimulatory signals, like B7/CD28 using CTLA4Ig, deeply impaired CD4+ T cell responses against herpes simplex virus and reduced immunoinflammatory lesions caused by HSV [78, 79]. Similar approach involving BTLA-4 is an inhibitory coreceptor that negatively affects lymphocyte activation. Systemic administration of recombinant plasmid DNA encoding BTLA (pBTLA) to the HSV-1 infected mice results in decrease of CD4+ T cells in infected cornea and reduced DTH response reducing HSV keratitis symptoms. pBTLA due to its immunosuppressive properties could be among future candidates used to prevent corneal scarring [80].

Therapeutic or prophylactic vaccine against ocular HSV-1 would be of significant value; it is still in the phase of preclinical experiments. Most HSV vaccines are focused on viral protein subunits mainly glycoprotein D (gD) and glycoprotein B (gB). The combination of DNA encoding five HSV-1 glycoproteins (gB, gC, gD, gE, gI) with DNA encoding IL-12p35 and Flt3L was proved to have better efficacy comparing to glycoproteins coding DNA alone that suggests a beneficial role of immunostimulatory adjuvants [81]. New data on the recognition of HSV by toll-like receptors might be used to further improve the future vaccines [81].

## 7. Conclusions

The HSV corneal disease is complex and involves the following components: the active infection itself, inflammation caused by active infection, and immune reaction to past infection. In primary infection, an inflammatory response triggers antigen-specific immune responses. CD4+ and CD8+ T cells are involved in modulation of infection and latency. Retained viral antigens may lead to subsequent immune-mediated stromal inflammation without viral replication. The HSV keratitis is the commonest cause of infectious blindness and available methods of treatment remain unsatisfactory. The novel insights into disease immunopathogenesis may allow for the development of more efficient therapeutical options based on immune interventions.
